# Wide distribution of autochthonous branched glycerol dialkyl glycerol tetraethers (bGDGTs) in U.S. Great Basin hot springs

**DOI:** 10.3389/fmicb.2013.00222

**Published:** 2013-08-08

**Authors:** Brian P. Hedlund, Julienne J. Paraiso, Amanda J. Williams, Qiuyuan Huang, Yuli Wei, Paul Dijkstra, Bruce A. Hungate, Hailiang Dong, Chuanlun L. Zhang

**Affiliations:** ^1^School of Life Sciences, University of Nevada, Las VegasLas Vegas, NV, USA; ^2^State Key Laboratory of Marine Geology and School of Ocean of Earth Sciences, Tongji UniversityShanghai, China; ^3^Department of Geology and Environmental Earth Science, Miami UniversityOxford, OH, USA; ^4^Department of Marine Sciences, University of GeorgiaAthens, GA, USA; ^5^Department of Biological Sciences and Center for Ecosystem Science and Society, Northern Arizona UniversityFlagstaff, AZ, USA

**Keywords:** geothermal springs, membrane-spanning lipids, bGDGTs, thermophiles, Great Basin, lipids

## Abstract

Branched glycerol dialkyl glycerol tetraethers (bGDGTs) are membrane-spanning lipids that likely stabilize membranes of some bacteria. Although bGDGTs have been reported previously in certain geothermal environments, it has been suggested that they may derive from surrounding soils since bGDGTs are known to be produced by soil bacteria. To test the hypothesis that bGDGTs can be produced by thermophiles in geothermal environments, we examined the distribution and abundance of bGDGTs, along with extensive geochemical data, in 40 sediment and mat samples collected from geothermal systems in the U.S. Great Basin (temperature: 31–95°C; pH: 6.8–10.7). bGDGTs were found in 38 out of 40 samples at concentrations up to 824 ng/g sample dry mass and comprised up to 99.5% of total GDGTs (branched plus isoprenoidal). The wide distribution of bGDGTs in hot springs, strong correlation between core and polar lipid abundances, distinctness of bGDGT profiles compared to nearby soils, and higher concentration of bGDGTs in hot springs compared to nearby soils provided evidence of *in situ* production, particularly for the minimally methylated bGDGTs I, Ib, and Ic. Polar bGDGTs were found almost exclusively in samples ≤70°C and the absolute abundance of polar bGDGTs correlated negatively with properties of chemically reduced, high temperature spring sources (temperature, H_2_S/HS^−^) and positively with properties of oxygenated, low temperature sites (O_2_, NO^−^_3_). Two-way cluster analysis and nonmetric multidimensional scaling based on relative abundance of polar bGDGTs supported these relationships and showed a negative relationship between the degree of methylation and temperature, suggesting a higher abundance for minimally methylated bGDGTs at high temperature. This study presents evidence of the widespread production of bGDGTs in mats and sediments of natural geothermal springs in the U.S. Great Basin, especially in oxygenated, low-temperature sites (≤70°C).

## Introduction

Glycerol dialkyl glycerol tetraethers (GDGTs) are core membrane-spanning lipids that resist delamination and impart membrane stability at high temperature and low pH. GDGTs are synthesized by a variety of extremophiles and some non-extremophiles and occur widely in nature (Schouten et al., 2000, 2013 and references therein). The isoprenoid GDGTs (iGDGTs) contain alkyl groups constructed by polymerization of isoprene subunits and are synthesized by physiologically and phylogenetically diverse archaea [reviewed in Schouten et al. (2013)]. A separate group of GDGTs, the branched GDGTs (bGDGTs), have been identified in lipid extracts prepared from a wide variety of environments, including peat bogs, soils, estuaries, and lake and river water and sediments (Schouten et al., 2000; Sinninghe Damsté et al., 2000, 2009; Weijers et al., 2006a, 2009; Zink et al., 2010; Zhang et al., 2012; Liu et al., 2013; Yang et al., 2011, 2013; Wang et al., 2013). bGDGT types vary according to the degree of methylation of the alkyl chains, with 4–6 methyl groups per GDGT, and the number of cyclopentyl moieties, with 0 to 3 rings per GDGT (Figure S1) (Sinninghe Damsté et al., 2000).

Although bGDGTs contain the membrane-spanning feature and ether linkages similar to archaeal iGDGTs, they are not isoprenoidal and differ in the stereochemical configuration of the second carbon position of the glycerol backbone; therefore, bGDGTs were proposed to have a bacterial rather than archaeal origin (Weijers et al., 2006b). Following a number of investigations focusing on microorganisms inhabiting water-saturated, anaerobic peat bog environments (e.g., Weijers et al., 2009), a single bGDGT, bGDGT I (Figure S1), was discovered in pure cultures of *Acidobacteria*, specifically *Edaphobacter aggregans* Wbg-1^*T*^ and *Acidobacteriaceae* strain A2 - 4c (Sinninghe Damsté et al., 2011). The origin of the other bGDGTs found in peat bogs and other natural environments (Figure S1) remains unknown. Yet-uncultivated *Acidobacteria* are candidates for these other bGDGTs since all tested *Acidobacteria* produce abundant 13,16-dimethyl octacosanedioic acid (*iso*-diabolic acid), a likely precursor in bGDGT synthesis (Sinninghe Damsté et al., 2011), and since various bGDGTs and yet-uncultivated *Acidobacteria* co-exist in peat soils (Weijers et al., 2009).

Although bGDGTs are known predominantly from soils, a few studies have documented bGDGTs in terrestrial geothermal environments. Substantial amounts of bGDGTs, accounting for up to 64% of total GDGTs, were recovered in all nine geothermal spring samples collected by Schouten et al. (2007) in Yellowstone National Park. However, the authors suggested that the majority of bGDGTs were from soil runoff (Schouten et al., 2007). A subsequent study of bGDGT lipids in soil transects near two springs in Surprise Valley, California, revealed the presence of bGDGTs in geothermally heated soils that were 12–41°C at the time of collection (Peterse et al., 2009). For both springs, bGDGT concentrations in heated soils near the springs were greatly elevated over cooler, more distant soils. bGDGTs were also recovered from two alkaline hot springs in Tibet, ranging from 52.0–83.6°C (He et al., 2012). The high concentrations of bGDGTs in some samples and a unique lipid profile compared with nearby soils were consistent with bGDGT production in the springs. Most recently, a study of GDGTs extracted from natural sediments and incubation experiments in Great Boiling Spring (62–82°C), Nevada, provided multiple lines of evidence demonstrating the *in situ* production of bGDGTs in this hot spring (Zhang et al., 2013). In addition, cellulosic material that was incubated *in situ* in both sediments and the water column at 77 and 85°C also contained significant concentrations of bGDGTs. The bGDGT data were compared with 16S rRNA gene pyrotag datasets obtained from the same samples (Cole et al., 2013; Peacock et al., 2013), revealing that *Acidobacteria* were rare in all samples (<0.01% of pyrotags), and suggesting that the bGDGTs were produced by thermophilic bacteria, possibly yet-uncultivated *Bacteroidetes*, candidate phylum EM3 (Rinke et al., 2013), or candidate phylum “*Atribacteria*” (formerly candidate phylum OP9; Dodsworth et al., 2013; Rinke et al., 2013).

In this study, we sought to augment the finding by Zhang et al. (2013) and to further test the hypothesis that bGDGTs are produced in natural geothermal environments and to examine relationships between bGDGT composition and abundance and physicochemical setting. To do this, we examined the distribution and abundance of bGDGTs in natural sediment and mat samples collected from a variety of geothermal springs in the northwest Great Basin (U.S.A.), along with a large physicochemical dataset. bGDGTs were widespread in the springs but were more abundant in cooler, more oxidized springs with well-developed microbial mats, defining the optimal habitat for bGDGT-producing thermophiles in habitats ≤70°C with abundant biomass.

## Materials and methods

### Sample sites and physicochemical measurements

**Figure 1:**
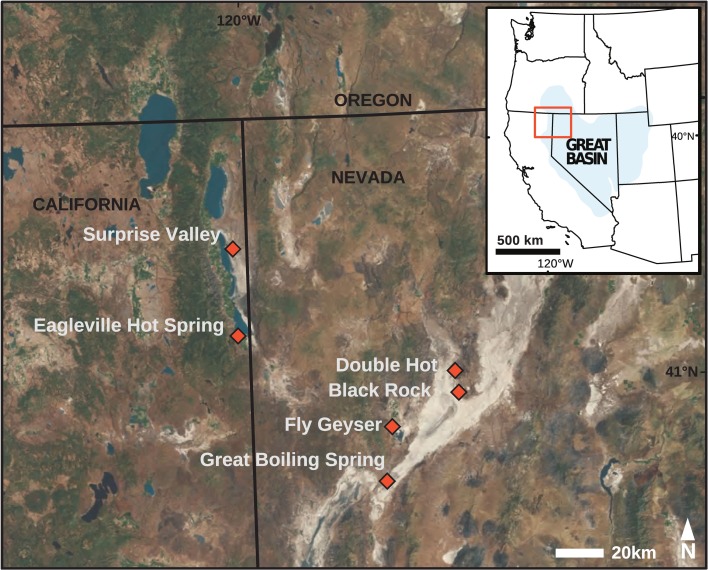
**The study area (inset, orange) was located with the U.S. Great Basin.** Hot spring sampling sites (orange diamonds) were located within California and Nevada.

Sediment and mat samples (top 1 cm of sediment/mat and water interface) were collected from eight hot springs located in northwest Nevada and northeast California (Figure 1). The same samples were used for simultaneous extraction of bGDGTs and iGDGTs; the results for iGDGTs are presented separately (Paraiso et al., in review). The sediment/mat water interface (top ~1 cm) was collected. All samples were frozen on dry ice in the field and were transported and stored frozen (−80°C) until they were thawed for lipid extraction and analysis. Field geochemical measurements and samples for laboratory geochemical measurements were taken prior to sediment and mat sampling (Costa et al., 2009; Vick et al., 2010). Briefly, temperature, pH, and conductivity were determined using a pH probe with temperature correction (LaMotte 5 Series, Chestertown, MD or YSI Model 30, Yellow Springs, OH and WTW Model pH330i, Weilheim, Germany). Redox-sensitive analytes (O_2_ and sulfide) were measured in the field using commercial kits (Hach, USA) with modifications for high temperature according to Miller-Coleman et al. (2012). Anions and cations were analyzed in the lab by ion chromatography (Dionex DX-500 chromatograph, AS14A column, with 10 μ M Na_2_CO_3_/NaHCO_3_ as an eluent, Dionex, USA) and direct current plasma emission spectrometry (DCP-OES, Beckman, USA). Dissolved inorganic nitrogen species (NO^−^_3_, NO^−^_2_, NH_3_/NH^+^_4_) were measured by automated colorimetry (Lachat, USA) as described by Dodsworth et al. (2011a,b).

### Lipid extraction and liquid chromatography-mass spectrometry (LC–MS)

bGDGTs and iGDGTs were extracted from 5 gram lyophilized samples using a modified Bligh-Dyer extraction method as detailed by Lengger et al. (2012). The resulting lipids were separated by silica-gel column chromatography using *n*-hexane:ethyl acetate (1:1, v:v) to collect the nonpolar fraction (F1) and MeOH to collect the polar (F2) fraction. The polar fraction was divided. One aliquot, fraction F2A was not processed further. The other fraction, F2B was hydrolyzed with MeOH:HCl (95:5; v:v) at 70°C for 3 h, extracted with DIH_2_O and DCM. All three fractions were dried under N_2_, dissolved in *n*-hexane:isopropyl alcohol (99:1; v:v), filtered through a 0.45 μm polytetrafluoroethylene filter, and dried under N_2_. Dried lipids were dissolved in 600 μL of *n*-hexane:isopropyl alcohol (99:1; v:v) for analysis. F1 and F2A were run directly on LC–MS and F2B was run on LC–MS after hydrolysis. F2A was to check whether any core GDGTs may be present in the polar fraction. If there are any core-GDGTs detected in F2A, they will be subtracted from F2B and added to F1.

Each fraction was spiked with a GDGT C_46_ internal standard and analyzed on an Agilent 1200 liquid chromatography equipped with an automatic injector coupled to QQQ 6460 MS and Mass Hunter LC-MS Manager software. Separation of GDGT peaks was achieved using a Prevail Cyano column (2.1 × 150 mm, 3 μm; Alltech, Deerfield, IL, USA) as described in Zhang et al. (2012) and GDGTs were quantified using Agilent 6460 triple-quadrupole MS with an atmospheric pressure chemical ionization (APCI) source as (Zhang et al., 2012).

### Statistical analyses

Two-way cluster analyses and non-metric multidimensional scaling (NMS) were completed in PC-ORD (MjM Software Design). Input data for these analyses included the relative abundance of the polar fractions of individual bGDGTs. Within PC-ORD, lipid data were normalized according to the maximum value of each lipid and were used to calculate a Sørensen (Bray-Curtis) distance matrix, which was applied in NMS and two-way clustering analyses. For two-way cluster analyses, the flexible beta method (β = −0.25) was used to create an agglomerative hierarchical clustering tree, with methods modified from those in Pearson et al. (2008). NMS was run in PC-ORD's “autopilot mode,” which determined the optimal number of axes through a Monte Carlo test of 100 runs (50 runs on actual data, 50 runs on random data). The final ordination included 99 runs, which were completed for the recommended number of axes. Environmental data were overlaid on the NMS plot to show correlation between lipids and geochemistry. Samples with no bGDGTs were removed from NMS and two-way cluster analyses to avoid the bias that may occur when a GDGT is assumed to be absent when, in fact, it may be present below detection concentrations.

Non-parametric Mann–Whitney tests, Spearman's rho correlation coefficients, and select linear regressions were executed in IBM SPSS Statistics 19 to identify relationships among the GDGTs and geochemical analytes. Analyses were completed at the 0.05 level of significance.

## Results

### Branched GDGTs in hot spring sediments and mats and adjacent soils

**Figure 2:**
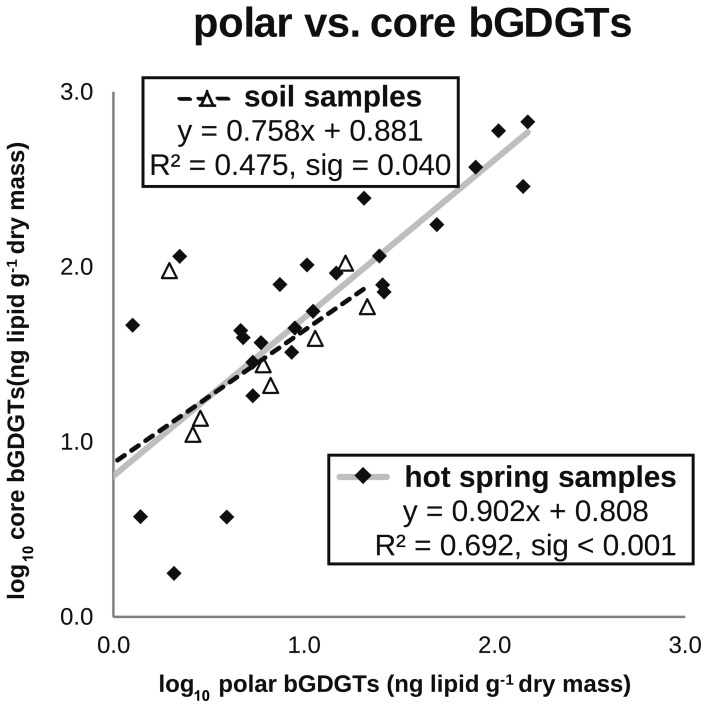
**Absolute abundance of polar bGDGTs vs. core bGDGTs for soil and hot spring samples.** Samples below the method detection limit for polar bGDGTs are not shown and were not used for in regression analyses (14 hot spring, 4 soil). Samples with <1 ng/g of polar or core iGDGTs, with negative log10 values are not shown; however, they were used for regression analyses (3 hot spring, 2 soil).

bGDGTs were detected in 38 of 40 hot springs sampled, spanning a temperature range of 31–95°C and a pH range of 6.8–10.7, across several distinct geothermal systems in the northwest Great Basin (Table 1; Figure 1; Table S1). Total bGDGT concentrations ranged up to 824 ng/g dry mass in hot springs (Table 1); lower concentrations were observed in adjacent desert soils (Table S1; Figure 2). A strong, positive, linear relationship between log-transformed core and polar bGDGTs was observed (*r*^2^ = 0.692, sig < 0.001). Similar log-linear relationships existed for individual bGDGTs in hot spring samples, particularly bGDGT I, Ib, Ic, II, and IIb (Figure S2); however, no such relationship was clear for bGDGT III, while polar bGDGT IIc, IIIb, and IIIc were not detected in any hot spring samples. Samples from desert soils adjacent to the springs showed a similar relationship between log-transformed core and polar bGDGTs (*r*^2^ = 0.475, sig = 0.040); however, significant linear relationships for individual lipids were only obtained for bGDGT I and bGDGT IIb (Figure S2).

**Table 1:** **Branched GDGT absolute abundance in hot spring samples**.

**Hot spring**	**Hot spring location site**	**Temperature °C**	**pH**	**Absolute abundance of core + polar bGDGTs (ng of lipid/g of dry mass)**										
				**bGDGT Ic m/z 1018**	**bGDGT Ib m/z 1020**	**bGDGT I m/z 1022**	**bGDGT IIc m/z 1032**	**bGDGT IIb m/z 1034**	**bGDGT II m/z 1036**	**bGDGT IIIc m/z 1046**	**bGDGT IIIb m/z 1048**	**bGDGT III m/z 1050**	**Total bGDGT**	**% Polar bGDGTs**
**GREAT BOILING SPRINGS**														
Great Boiling Springs	GBS 19	95.00	6.80	8.98	25.42	45.25	1.42	7.03	20.94	BDL	1.24	6.94	117.22	1.9
	GBS A	80.90	7.41	BDL^a^	BDL	BDL	BDL	BDL	BDL	BDL	BDL	BDL	BDL	N/A^b^
	GBS B	77.50	7.35	0.04	0.14	0.44	BDL	0.04	0.11	BDL	BDL	0.05	0.82	0.00
	GBS C	65.00	7.69	BDL	1.31	6.15	BDL	0.58	1.34	BDL	BDL	BDL	9.38	0.00
	GBS 61 - Y	60.90	8.00	34.03	51.04	328.31	BDL	0.90	10.44	BDL	BDL	5.09	429.81	33
Sandy's Springs West	SSW SOURCE	80.00	7.37	0.67	1.07	2.98	BDL	0.69	3.28	BDL	BDL	3.50	12.19	0.00
	SSW 70	69.50	7.86	1.14	2.85	30.10	BDL	0.68	2.89	BDL	BDL	3.42	41.08	21
	SSW 60	59.50	7.90	69.37	100.52	445.84	1.58	11.07	52.81	BDL	BDL	23.05	704.24	15
	SSW 50 (OF)	50.50	8.17	BDL	52.08	197.57	1.66	9.95	5.56	BDL	BDL	BDL	266.82	7.8
	SSW 40	39.40	8.49	62.23	217.09	487.92	BDL	4.66	40.53	BDL	BDL	11.40	823.83	18
Rick's Hot Creek	RHC 5	90.20	7.49	0.18	0.19	5.04	BDL	0.59	3.01	BDL	0.33	2.20	11.54	0.00
	RHC 4	78.60	7.75	0.29	0.56	1.73	BDL	0.41	0.74	BDL	BDL	0.37	4.10	0.00
	RHC 3	69.40	7.96	0.05	0.07	1.50	BDL	0.06	0.37	BDL	0.06	0.34	2.45	23
	RHC 2	61.50	8.23	0.66	1.81	4.19	BDL	0.10	0.61	BDL	0.04	0.25	7.66	52
	RHC 1	52.00	8.43	5.07	9.87	53.03	BDL	3.75	19.76	BDL	2.18	13.07	106.73	14
**OTHER BLACK ROCK DESERT SPRINGS**														
Fly Geyser	FG 60	60.00	8.37	BDL	BDL	BDL	BDL	BDL	BDL	BDL	BDL	BDL	BDL	N/A
	FG 50	50.00	8.60	0.45	0.91	3.68	BDL	BDL	0.09	BDL	BDL	BDL	5.13	27
	FG 40	42.00	8.80	BDL	1.11	1.80	BDL	0.23	0.44	0.20	0.07	BDL	3.85	54
Double Hot Springs	DH SOURCE	79.60	8.05	0.53	1.05	2.43	0.18	BDL	0.61	BDL	BDL	0.18	4.98	0.00
	DH 70	69.60	8.37	2.37	4.39	26.32	BDL	1.29	10.18	BDL	BDL	3.32	47.87	9.7
	DH 60	59.90	8.74	3.07	5.25	13.01	BDL	0.41	1.63	BDL	BDL	0.37	23.74	23
	DH SOURCE 2	54.50	8.50	5.75	16.91	17.76	BDL	0.69	2.74	BDL	BDL	0.39	44.24	11
	DH 50	49.90	9.09	BDL	13.09	28.12	BDL	BDL	1.60	BDL	BDL	BDL	42.81	14
	DH 43	43.50	9.24	3.81	35.06	48.83	0.33	4.85	9.39	BDL	0.50	1.94	104.71	25
	DH 31	31.00	10.70	BDL	BDL	0.23	BDL	0.08	0.48	BDL	BDL	0.12	0.91	0.00
Black Rock	BR 45	45.50	7.88	6.20	70.02	92.20	0.68	7.56	32.27	BDL	0.68	14.86	224.47	225
**SURPRISE VALLEY SPRINGS**														
Eagleville	EV 44	43.80	9.73	14.58	35.60	33.69	0.66	0.76	8.29	BDL	BDL	4.70	98.28	9.2
	EV 43	43.80	9.73	20.03	177.94	199.25	1.98	27.67	21.86	BDL	BDL	1.69	450.42	27
	SV SOURCE	86.00	8.34	1.31	2.42	7.62	0.15	0.31	1.25	BDL	BDL	0.20	13.26	18
Surprise Valley	SV 70	69.20	8.50	2.86	7.80	20.50	0.62	3.46	7.88	0.09	0.32	4.18	47.71	0.00
	SV 60	59.60	8.64	4.50	20.41	41.33	0.41	3.17	12.80	BDL	0.28	3.87	86.77	27
	SV 50	48.50	8.78	5.69	29.27	87.53	0.40	3.28	11.68	BDL	0.43	1.96	140.24	18
	SV 40	41.30	9.07	1.23	19.23	65.25	BDL	4.53	19.32	BDL	BDL	3.28	112.84	0.00
	SVX SOURCE	83.70	8.41	0.56	2.18	BDL	3.37	0.67	1.44	BDL	BDL	13.60	21.82	2.6
	SVX 70	68.50	8.58	1.26	8.66	21.30	BDL	0.73	0.70	BDL	BDL	1.25	33.90	8.6
	SVX 60	60.60	8.72	5.42	20.86	35.61	BDL	0.90	3.49	BDL	BDL	0.57	66.85	18
	SVX 50	50.00	8.98	3.05	23.67	22.86	0.23	0.67	2.59	BDL	BDL	0.59	53.66	9.2
	SVX 2	83.40	8.24	6.54	15.66	90.26	0.61	2.12	13.01	BDL	BDL	1.96	130.16	0.00
	SVX 1	76.50	8.32	0.67	1.11	3.68	0.08	0.24	1.18	BDL	BDL	BDL	6.96	16
	SVX 3	40.80	8.22	2.97	24.02	32.08	0.60	5.84	13.02	BDL	BDL	3.61	82.14	0.00

a BDL, below detection limit; detection limit for core lipids is 0.8 pg.

b N/A, percent polar lipids not available because total polar + core bGDGTs were below detection limit.

**Figure 3:**
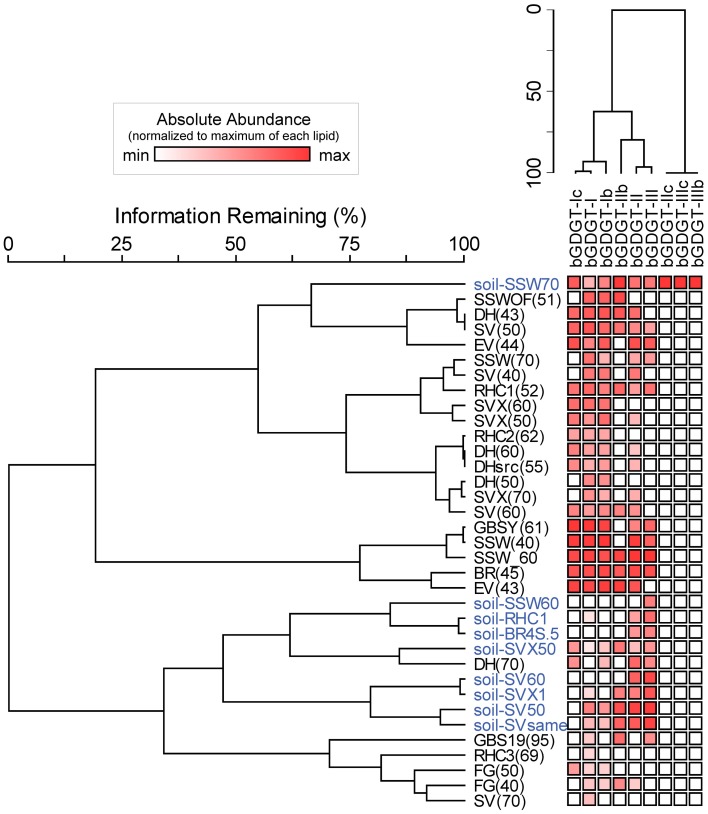
**Two-way cluster analysis organizes hot spring samples (black) and soil samples (blue) according to similarity in polar bGDGT composition (horizontal dendrogram).** bGDGTs are organized according to their presence and abundance in the samples (vertical dendrogram). Heat map colors indicate minimum (white) to maximum (red) abundance of bGDGTs, wherein colors are scaled by each lipid's maximum relative abundance in hot spring and soil samples.

bGDGT I, Ib, and Ic were present in many hot spring samples at concentrations of one order of magnitude or more higher than in adjacent soils (Figure S2). A two-way cluster analysis based on the relative abundance of individual polar bGDGTs showed that hot spring samples had distinct bGDGT composition compared with soil samples, with bGDGT I, Ib, and Ic, in decreasing order, being the most common and abundant bGDGTs in hot spring samples and bGDGT II and III being the most common and abundant bGDGTs in soil samples (Figure 3).

### Relationship between BGDGT abundance and temperature in hot spring samples

**Figure 4:**
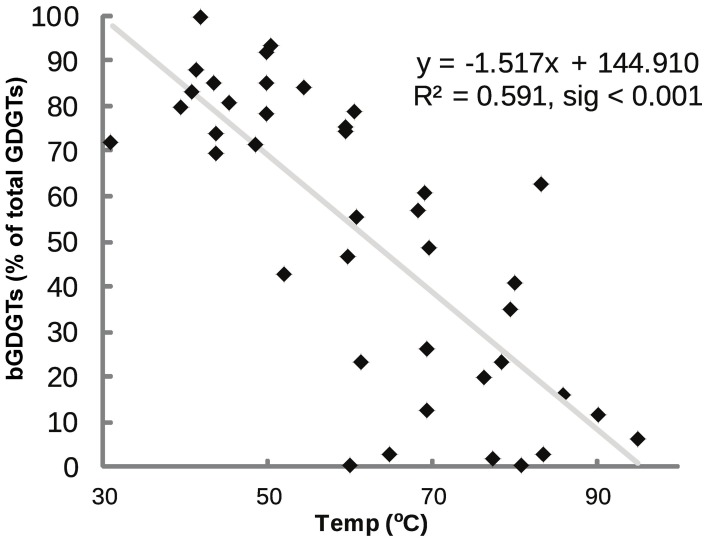
**Percent bGDGTs (core plus polar) vs. temperature.** Percent bGDGTs was calculated by dividing total bGDGTs by the sum of all GDGTs (iGDGTs + bGDGTs).

bGDGTs were the dominant GDGTs in some samples, comprising up to 99.5% of total GDGTs (bGDGTs plus iGDGTs), whereas iGDGTs dominated other samples, with bGDGTs comprising <1% (Figure 4). The relative abundance of bGDGTs and iGDGTs was related to temperature, with bGDGTs abundant at lower temperatures and iGDGTs dominating at higher temperatures (*r*^2^ = 0.591, sig < 0.001; Figure 4).

**Figure 5:**
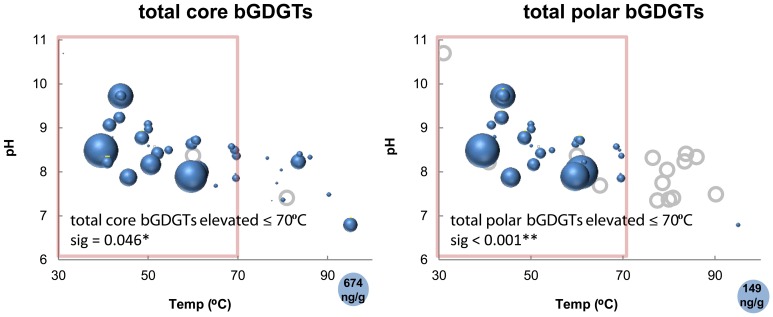
**Absolute abundance of core and polar bGDGTs as a function of temperature and pH.** Bubble areas (blue) are proportional to the absolute abundance of the core and polar bGDGTs. Circle in lower right corner represents the maximum absolute abundance in ng lipid g-1 dry mass. Gray circles represent zero abundance. Pink boxes delimit 70°C, at and below which bGDGTs are nearly ubiquitous in Great Basin hot springs. Statistical data refer to Mann–Whitney tests demonstrating the significantly higher abundance of bGDGTs ≤70°C.

The absolute abundance of bGDGTs was also related to temperature, with most samples >70°C containing relatively low concentrations of core bGDGTs (≤62 ng/g; Figure 5). This pattern was even clearer for polar bGDGTs, which are unstable in the environment, and therefore, generally interpreted to represent living biomass (White et al., 1979). Polar bGDGTs were absent in all but one sample that was >70°C at the time of collection (Figure 5). A Mann–Whitney test confirmed the significance of 70°C as a cutoff for both core and polar bGDGT distribution within this dataset (core sig = 0.046; polar sig < 0.001). Spearman's rank correlation analysis relating the absolute abundance of polar bGDGTs and geochemical measurements revealed significant negative relationships between polar bGDGT abundance and temperature (ρ = −0.582; sig < 0.001) and sulfide concentration (ρ = −0.405; sig = 0.014), and significant positive relationships between bGDGT abundance and dissolved oxygen (ρ = 0.508; sig = 0.001) and nitrate (ρ = 0.650; sig < 0.001) concentrations. Spearman's rank correlation analysis with the absolute abundance of polar bGDGT I, Ib, and Ic, analyzed separately, revealed similar relationships (Table S2). In contrast, the absolute abundance of other polar bGDGTs had few significant correlations with geochemical measurements (Table S2).

The sample from the highest temperature in the Great Boiling Spring geothermal field, GBS-19, contained a low concentration of polar bGDGTs (2.2 ng/g dry mass), but the highest concentrations of bGDGT IIb (0.80 ng/g dry mass) and bGDGT III (0.80 ng/g dry mass) among hot spring samples.

### Co-variation between BGDGTs in hot spring samples

**Figure 6:**
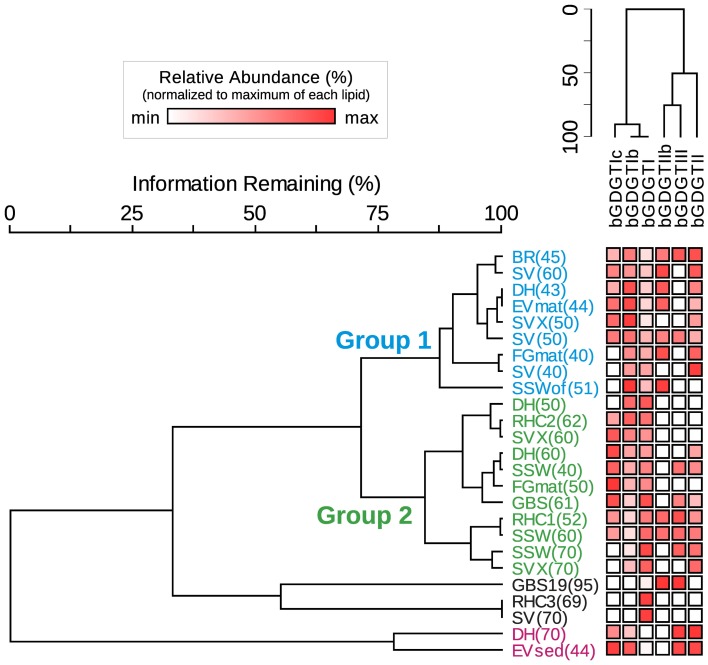
**Two-way cluster analysis organizes hot spring samples according to similarity in polar bGDGT composition (horizontal dendrogram).** bGDGTs are organized according to their presence and abundance in the samples (vertical dendrogram). Heat map colors indicate minimum (white) to maximum (red) abundance of bGDGTs, wherein colors are scaled by each lipid's maximum relative abundance in hot spring samples. Maximum relative abundance (%) of polar lipids is as follows: bGDGT-Ic (21.5), bGDGT-Ib (45.4), bGDGT-I (100.0), bGDGT-IIb (35.7), bGDGT-II (56.0), bGDGT-III (28.6).

bGDGT I, bGDGT Ib, and bGDGT II, in order of decreasing abundance, were the dominant bGDGTs in hot spring samples (Table 1), comprising >70% of all polar bGDGTs in all samples except for GBS-19. A two-way cluster analysis based on the relative abundance of individual polar bGDGTs was created to group geothermal springs and to examine the co-occurrence of individual bGDGTs in the environment (Figure 6). The cluster analysis along the horizontal axis revealed two major groups of samples. Group 1 included most low-temperature spring samples, with all except one at ≤51°C. Group 2, in contrast, included sites ranging up to 70°C, with all except one collected from ≥50°C. Several other sites, mostly ≥70°C, had more unique bGDGT profiles and clustered separately. No geographical clustering was apparent, consistent with non-significant results in the Spearman's rank correlation analysis for the majority of chemical analytes, particularly redox-inactive species. The cluster analysis along the y-axis showed that the minimally methylated tetraethers, bGDGT I, bGDGT Ib, and bGDGT Ic, co-varied, leaving a second, less tight cluster for the more highly methylated tetraethers bGDGT II, bGDGT IIb, and bGDGT III. A heatmap associated with the two-way cluster analysis generally supported both cluster analyses and allows visualization of the distribution of each individual polar lipid within the dataset.

**Figure 7:**
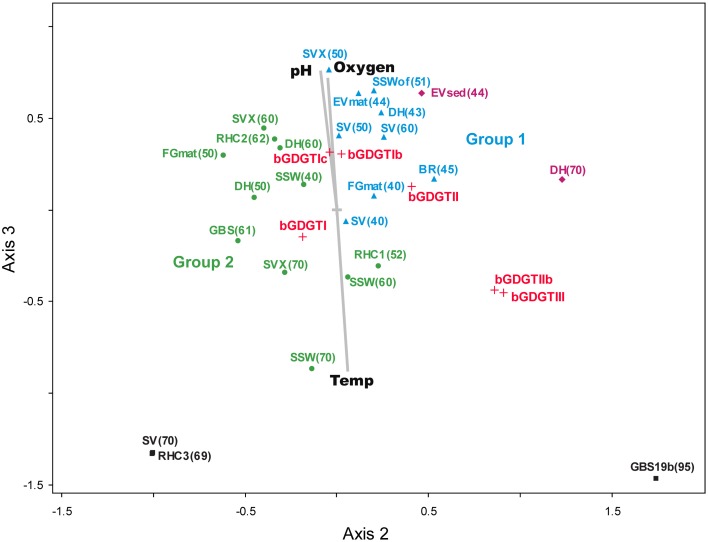
**The NMS plot shows relationships among polar bGDGTs (relative abundance) and environmental variables.** Samples (solid shapes) are arranged accordingly to similarity in polar bGDGT composition. Distance on the plot is proportional to dissimilarity in lipid composition. Lipids (red +) appear where the centroid of that variable would be placed within the NMS model. Environmental variables (gray) appear as vectors that indicate relative correlation with NMS axes. Colors of samples sites correspond to groups identified in the two-way cluster analysis.

Multivariate relationships between the relative abundance of individual, polar bGDGTs and physicochemical factors were examined using nonparametric multidimensional scaling (NMS; Figure 7). In the NMS plot shown here, distance is proportional to multivariate dissimilarity in lipid composition (as calculated by a Sørensen distance matrix). Samples that are highly related are plotted closely together, while bGDGT types are plotted according to where the centroid of each lipid variable would occur. Physicochemical analytes are plotted as vectors, wherein the length and direction of a vector is proportional to its relative correlation with NMS axes.

The 3-dimensional NMS model was a low-stress and statistically significant ordination (min stress = 5.921, *p* = 0.0196), which suggested strong underlying structure in lipid composition. The NMS plot supported Group 1 and Group 2 identified by two-way cluster analysis and indicated a strong correlation between the lipid-derived NMS model and temperature (*r*^2^ = 0.438, correlation with NMS axis 3). Other strong correlations with the lipid-derived NMS model were oxygen (*r*^2^ = 0.386, axis 3) and pH (*r*^2^ = 0.384, axis 3), both of which co-vary negatively with temperature in alkaline geothermal systems (Nordstrom et al., 2005). Individual bGDGTs were spread along a line originating near the center of the plot, populated by the minimally methylated bGDGT I, bGDGT Ib, and bGDGT Ic, and terminating in the lower right quadrant with the highly methylated bGDGT III. The trend along axis 3 was in the same direction as the temperature vector and opposite to the pH and oxygen vectors, demonstrating a possible positive relationship between methylation and temperature and covariates of temperature; however, this trend was opposite the pattern of bGDGT I, bGDGT Ib, and bGDGT Ic dominating in most hot spring samples and may have been driven by GBS 19, which was an unusual sample that was dominated by bGDGT IIb and bGDGT III.

## Discussion

The abundance of bGDGTs in geothermal samples reported here is similar to those in geothermally heated soils at hot spring margins ranging from 18 to 41°C (9–2,400 ng/g dry mass; Peterse et al., 2009) and in hot spring sediments in Great Boiling Spring (GBS), Nevada, ranging from 62 to 82°C (15–335 ng/g dry mass; Zhang et al., 2013) and Gulu and Yangbajing hot springs in Tibet, ranging from 52 to 83.6°C (up to 235 ng/g dry mass; He et al., 2012). These bGDGT concentrations are also similar to those in non-thermal soils, such as those throughout the Pearl River watershed (10–770 ng/g soil; Zhang et al., 2012) but much lower than some terrestrial environments such as decaying peat (up to 80 μg/g dry mass; Weijers et al., 2006b).

Our data here support the *in situ* production of bGDGTs in some Great Basin hot springs based on four lines of evidence: (1) the widespread distribution of bGDGTs in Great Basin geothermal springs, (2) the higher concentration of bGDGTs in many ≤70°C hot spring samples as compared with nearby desert soils, (3) the strong relationship between core and polar bGDGTs in geothermal samples (Liu et al., 2011), and (4) the distinctiveness of soil and hot spring bGDGT profiles (Figure 3). These trends were particularly clear for bGDGT I, Ib, and Ic, providing strong evidence for their autochthonous production in the springs. However, some hot springs, particularly those >70°C had substantially lower abundance of bGDGTs than surrounding soils, particularly polar bGDGTs (Figures 2, 5; Table S1). These observations are consistent with those reported in Schouten et al. (2007) and suggest that soil runoff may be a dominant source of bGDGTs in hot springs at temperatures >70°C. However, the predominance of autochthonous bGDGTs in lower temperature springs is consistent with evidence that bGDGTs can also be produced in other aquatic systems such as lakes (Sinninghe Damsté et al., 2009; Tierney et al., 2010; Sun et al., 2011; Wang et al., 2012), rivers and estuaries (Zhu et al., 2011; Zhang et al., 2012), and geothermal springs (Zhang et al., 2013).

All data point to temperature as a possible factor controlling bGDGT production and suggest that most bGDGT-producing thermophiles are moderate thermophiles rather than hyperthermophiles. However, the effect of temperature on bGDGT production may be indirect, since incubation of lignocellulosic materials at 77 and 85°C in GBS led to *in situ* production of bGDGTs (Zhang et al., 2013). It is possible that bGDGT-producing thermophiles are members of consortia involved in the decomposition of complex biomass, which may only be found at high temperature on rare occasions, such as after delivery of allochthonous plant material into the spring or during degradation of photosynthetic mats after temperature up-shifts. The latter scenario could explain the variable presence of polar bGDGTs in >70°C sediments in GBS when it was sampled on different dates (Table 1; Zhang et al., 2013). Careful temperature monitoring and temporal sampling would be required to examine this possibility more rigorously. The temporal variability of the GBS geothermal field has been documented (Anderson, 1978) and is consistent with our own, more recent observations (e.g., Cole et al., 2013; Peacock et al., 2013). Although polar bGDGTs were present at low concentrations in GBS 19, which has a stable source at 95°C, we urge caution interpreting that bGDGTs can be synthesized at that temperature since that site has a steep geothermal gradient and therefore it is difficult to infer the precise temperature at which lipids may have been produced. Furthermore, this is currently the only report of polar bGDGTs at temperatures >85°C and the predominant lipids in that sample are heavily methylated, which is unusual in our dataset.

A few other geochemical measurements correlated with absolute or relative bGDGT abundance: nitrate, sulfide, oxygen, and pH, in addition to temperature. Although a direct effect of these variables on bGDGT-producing bacteria cannot be ruled out, we caution against using these data to infer details about the specific habitat and physiology of bGDGT-producing bacteria. These analytes co-vary with temperature in many geothermal outflows due to oxidation of reduced compounds present in the source water and gas (e.g., CO_2_) equilibration with the atmosphere (e.g., Nordstrom et al., 2005; Swingley et al., 2012) and their relationship to bGDGT composition and abundance is difficult to infer.

The absence of correlations between bGDGT abundance and any other geochemical analytes and the absence of biogeographic clusters in two-way cluster analysis and NMS plots (Figures 6, 7; Table S3) suggests that geological setting, and the resulting aqueous and solid-phase geochemistry, are not primary drivers of bGDGT production within Great Basin hot springs. For example, no apparent clusters were formed by geographically distinct springs that are classified as Na-Cl springs (Great Boiling Spring, Sandy's Spring West, Rick's Hot Creek) nor Na-HCO^−^_3_-Cl springs (Surprise Valley, Fly Geyser) (Anderson, 1978; Miller-Coleman et al., 2012). Our data are not consistent with a role of bGDGTs in stabilizing membranes in acidic conditions in these springs. In fact, bGDGT concentrations were directly proportional to pH, which is the opposite of what would be predicted if bGDGTs were serving a role to protect against low pH. This result does not rule out possible controls of geochemistry in bGDGT production in volcanically active geothermal systems such as Yellowstone National Park or the Rehai Geothermal Field in Tengchong, China, each of which host springs with a much wider pH range than the Great Basin (Brock, 1978; Zhang et al., 1987).

The wide range in the relative abundance of bGDGTs and iGDGTs in these springs (Figure 4) demonstrates great differences in relative habitability for bGDGT-producing bacteria and iGDGT-producing archaea. The inverse relationship between bGDGT/iGDGT ratios and temperature (Figure 4) is in agreement with general trends favoring archaea at environmental extremes; an increase in the relative abundance of archaea along temperature transects within GBS has been documented previously (Cole et al., 2013). The dominance of minimally methylated bGDGT I, bGDGT Ib, and bGDGT Ic among bGDGTs in this dataset is similar to the dominance of these three bGDGTs at higher temperatures in GBS (Zhang et al., 2013) and Yellowstone hot springs (Schouten et al., 2007). Our results are also consistent with the high abundance of bGDGT II and III in geothermal soils in Surprise Valley and suggest that those bGDGTs may be produced by soil microorganisms (Peterse et al., 2009). Tibet hot springs had bGDGT III as the dominant bGDGT (He et al., 2012). These results suggest that factors other than temperature may have some influence on the relative abundance of individual bGDGTs with different degree of methylation in geothermal ecosystems. The spreading of individual bGDGTs along axis 2 of the NMS plot (Figure 7), perpendicular to the temperature vector, also suggests factors other than temperature controlling degree of methylation.

## Conclusions

The results presented here, along with previous studies (Schouten et al., 2007; Peterse et al., 2009; He et al., 2012; Zhang et al., 2013) provide evidence for the production of bGDGTs by thermophilic bacteria inhabiting geothermal ecosystems, particularly bGDGT I, Ib, and Ic. Although bGDGTs can be produced up to 85°C (Zhang et al., 2013), the optimal conditions for bGDGT-producing microorganisms appears to be habitats ≤70°C, including both heated soils at hot spring margins and in sediments and mats within the springs themselves. As such, many bGDGT-producing bacteria appear to be moderate thermophiles, rather than hyperthermophiles. Temperature may be a master controller of the abundance of GDGTs in thermal springs, with moderate temperatures favoring a high relative abundance of bGDGTs and high temperature favoring high relative abundance of iGDGTs. However, the effect of temperature on bGDGT production may be indirect reflecting the presence of complex organic materials as incubation of lignocellulosic material at high temperatures led to production of bGDGTs at 77 and 85°C (Zhang et al., 2013). In contrast, geochemistry did not have a strong effect on bGDGT production, at least within the context of this study; however, other datasets, such as those from Tibetan hot springs, suggest global patterns of bGDGTs in geothermal systems may be more complex (He et al., 2012; Wang et al., unpublished data; Li et al., unpublished data).

### Conflict of interest statement

The authors declare that the research was conducted in the absence of any commercial or financial relationships that could be construed as a potential conflict of interest.
