# Supplementary material for: Wide distribution of autochthonous branched glycerol dialkyl glycerol tetraethers (bGDGTs) in U.S. Great Basin hot springs
Source: Front Microbiol. 2013 Aug 8;4:222. doi: 10.3389/fmicb.2013.00222 (PMC3737515; doi:10.3389/fmicb.2013.00222)
Supplement: Supplementary file 1 [file DataSheet1.PDF]

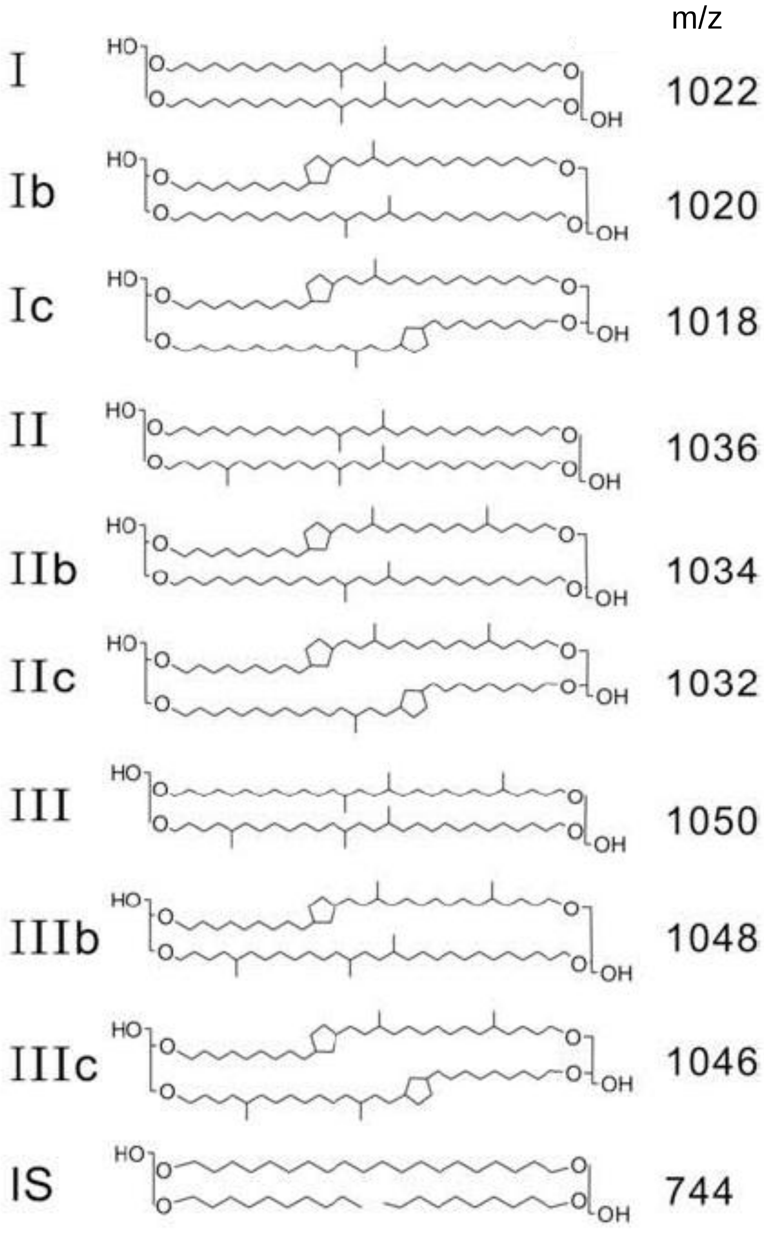

Figure S1. Schematic structures of branched GDGTs showing different numbers of methyl groups and cyclopentyl rings. IS, internal standard, C46 GDGT.
