# Supplementary material for: Wide distribution of autochthonous branched glycerol dialkyl glycerol tetraethers (bGDGTs) in U.S. Great Basin hot springs
Source: Front Microbiol. 2013 Aug 8;4:222. doi: 10.3389/fmicb.2013.00222 (PMC3737515; doi:10.3389/fmicb.2013.00222)
Supplement: Supplementary file 2 [file DataSheet2.PDF]

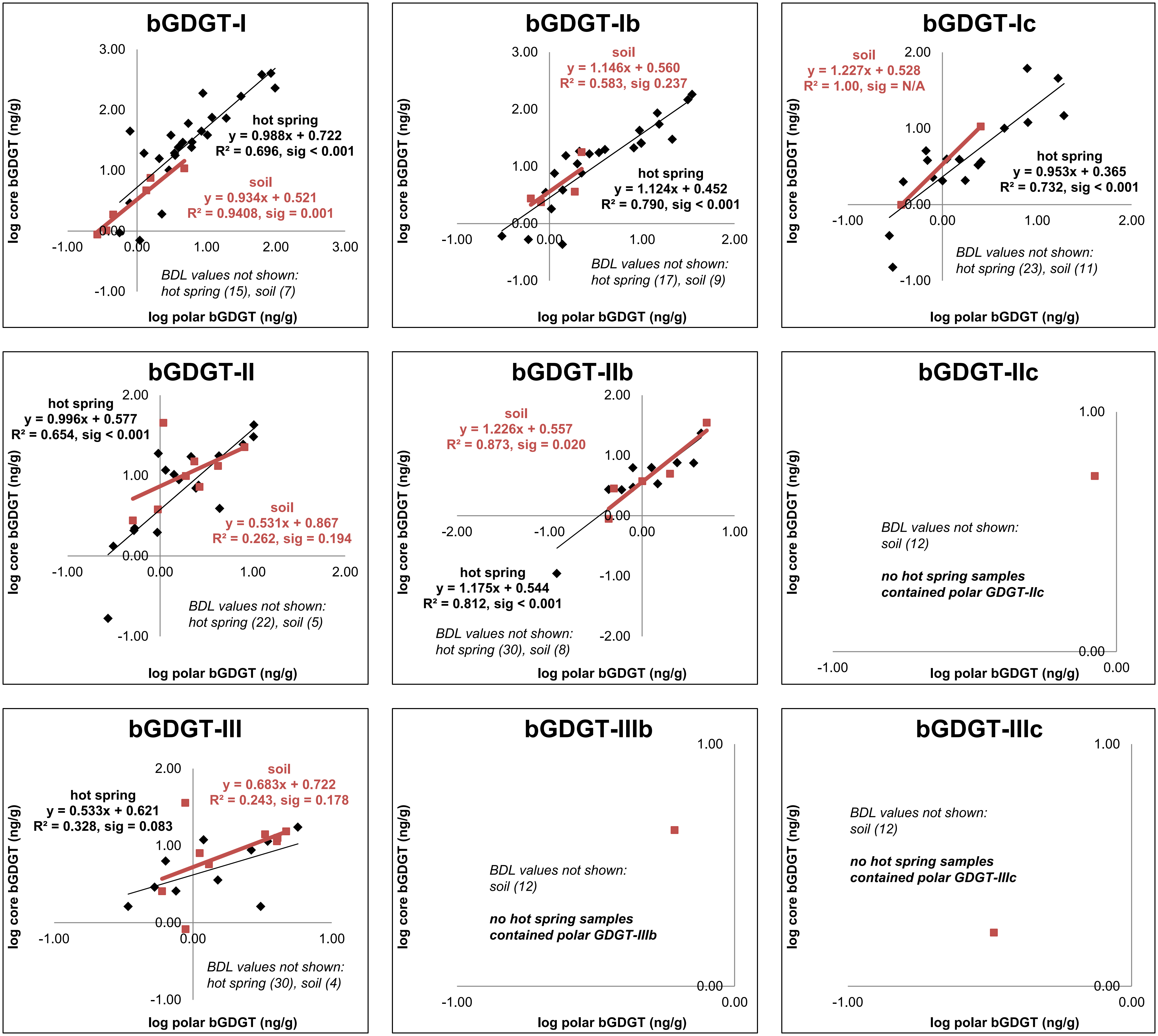

Figure S2: Absolute abundance of log-transformed polar versus core bGDGTs from soil (red) and hot spring (black) samples. Samples below the method detection limit (BDL) for polar bGDGTs are not shown and were not used for in regression analyses (missing values indicated in each plot). Lipid abundance is reported as ng lipid g^{-1} dry mass.
